# Reducing Persistent Postoperative Pain and Disability 1 Year After Breast Cancer Surgery: A Randomized, Controlled Trial Comparing Thoracic Paravertebral Block to Local Anesthetic Infiltration

**DOI:** 10.1245/s10434-013-3334-6

**Published:** 2013-10-29

**Authors:** Michelle Chiu, Gregory L. Bryson, Anne Lui, James M. Watters, Monica Taljaard, Howard J. Nathan

**Affiliations:** 1 Department of Anesthesiology, University of Ottawa, Ottawa, ON Canada; 2 Department of Surgery, University of Ottawa, Ottawa, ON Canada; 3 Clinical Epidemiology Program, Department of Epidemiology and Community Medicine, Ottawa Hospital Research Institute, University of Ottawa, Ottawa, ON Canada; 4 The Ottawa Hospital, Ottawa, ON Canada

## Abstract

**Background:**

The objective of this study was to compare the effect of thoracic paravertebral block (TPVB) and local anesthetic (LA) on persistent postoperative pain (PPP) 1 year following breast cancer surgery. Secondary objectives were to compare the effect on arm morbidity and quality of life.

**Methods:**

Women scheduled for elective breast cancer surgery were randomly assigned to either TPVB or LA followed by general anesthesia. An NRS value of >3 at rest or with movement 1 year following surgery defined PPP. Blinded interim analysis suggested rates of PPP much lower than anticipated, making detection of the specified 20 % absolute reduction in the primary outcome impossible. Recruitment was stopped, and all enrolled patients were followed to 1 year.

**Results:**

A total of 145 participants were recruited; 65 were randomized to TPVB and 64 to LA. Groups were similar with respect to demographic and treatment characteristics. Only 9 patients (8 %; 95 % CI 4–14 %) met criteria for PPP 1 year following surgery; 5 were in the TPVB and 4 in the LA group. Brief Pain Inventory severity and interference scores were low in both groups. Arm morbidity and quality of life were similar in both groups. The 9 patients with PPP reported shoulder-arm morbidity and reduced quality of life.

**Conclusions:**

This study reports a low incidence of chronic pain 1 year following major breast cancer surgery. Although PPP was uncommon at 1 year, it had a large impact on the affected patients’ arm morbidity and quality of life.

Breast cancer is the most common cancer among adult women.^1^ Pain in the ipsilateral arm and shoulder persisting 6 months or more after surgical treatment has been reported in 25–60 % of women.^2^–^5^ The etiology of this pain is multifactorial: The intercostobrachial nerve may be damaged in 80–100 % of patients undergoing axillary lymph node dissection (ALND), phantom and neuropathic pain may result from injury to nerves that supply the breast and axilla, and neuromas may form in scar tissue.^2^–^4^ Persistent postoperative pain (PPP) causes both psychological distress and disability in those affected.^6^,^7^ Reduced range of motion of the shoulder, muscle weakness, and lymphedema are also commonly reported.^6^–^8^

Multimodal analgesia strategies may reduce the incidence of PPP.^4^,^9^ Thoracic paravertebral nerve block (TPVB) and infiltration of local anesthetics (LA) at the surgical site are 2 techniques shown to independently reduce both short-term and long-term pain following breast surgery.^9^–^12^ The comparative impact of these techniques on PPP, arm morbidity, functional recovery, and quality of life has not been explored.

The objective of this study was to compare the effect of TPVB and LA on PPP 1 year following breast cancer surgery. We hypothesized that fewer women receiving TPVB would report numeric pain scores >3 1 year following surgery than those receiving LA. Secondary objectives of this study were to determine the effect of TPVB and LA on arm morbidity and quality of life at 1 year.

## Methods

This randomized, controlled, double-blinded trial was registered on ClinicalTrials.gov (NCT01089933) and approved by The Ottawa Hospital Research Ethics Board (Protocol 2006711-01H). It was conducted at The Ottawa Hospital, a 900-bed, tertiary care academic health science center affiliated with the University of Ottawa.

### Population

Women >18 years of age with breast cancer scheduled for elective breast conserving surgery with ALND, simple mastectomy with sentinel lymph node biopsy (SLNB), modified radical mastectomy (MRM), or ALND alone were evaluated by research personnel for participation. Exclusion criteria included ASA class 4 or 5, allergy to study medications, contraindications to TPVB, consumption of >20 mg of oral morphine or equivalent for >7 days, creatinine clearance <40 ml/minute (calculated using the Cockroft–Gault formula), preoperative radiation therapy or <100° of shoulder abduction or flexion.

### Randomization and Allocation Concealment

Eligible, consenting participants were allocated in parallel to TPVB and LA groups in a 1:1 ratio using computer-generated random numbers. Randomization was blocked in groups of 4–8 and stratified according to type of surgery. Allocation to TPVB or LA group was printed on cards placed in sealed, opaque, sequentially numbered envelopes. Envelopes were opened immediately before surgery by the attending anesthesiologist who prepared study medications. Patients, surgeons, and study personnel remained blinded to group allocation.

### Preoperative Management

All patients received oral celecoxib 400 mg and acetaminophen 650 mg 2 h preoperatively. Participants allocated to the TPVB group received nerve blocks at the T1–T6 levels; 5 mL of 0.5 % ropivacaine with epinephrine was injected at each space.^13^ Those allocated to the LA group received subcutaneous injections of 0.9 % NaCl at each level.

### Intraoperative Management

A standardized general anesthetic using propofol, fentanyl, dexamethasone, and volatile anesthetic gas was given. At the conclusion of surgery, the surgeon infiltrated the wound edges with 10 mL of study solution: 0.9 % NaCl in the TPVB group or 0.5 % ropivacaine with epinephrine in the LA group. After closure of the wound, a further 20 ml of study solution was instilled through the surgical drain, which was then clamped for 30 min.

### Postoperative Management

Following discharge from hospital, patients received oral acetaminophen 650 mg every 4 h for 48 h and celecoxib 200 mg every 12 h for 7 days in addition to their standardized hydromorphone opioid prescription.

### Outcome Assessments

All measures were compliant with the Initiative on Methods, Measurement, and Pain Assessment in Clinical Trials (IMMPACT) and were performed before and 1 year after surgery.^14^ Assessments were scheduled to not coincide with chemotherapy or radiation therapy.

#### Primary Outcome

Pain was assessed using an 11-point numeric rating scale (NRS) with 0 representing “no pain” and 10 representing “pain as bad as you can imagine.” An NRS value of >3 at rest or with movement 1 year following surgery defined PPP.

#### Secondary Outcomes

The Brief Pain Inventory (BPI) quantified the intensity of pain using four 11-point NRS scores that defined current, worst, least, and average pain scores over the preceding 24 h.^15^ The BPI also assessed the degree to which pain interferes with 7 daily activities using 11-point NRS scores anchored at 0 “does not interfere” and 10 “interferes completely.” Patients found to have PPP were referred to the Ottawa Regional Cancer Centre Pain and Symptom Management Clinic.

Flexion, extension, abduction, internal, and external rotation of the shoulder was assessed in both shoulders with a fixed scapula using a 12-in. universal goniometer. Relative shoulder movement, defined as ipsilateral movement/contralateral movement × 100, was assessed. A value of <90 % was taken to indicate functional impairment.^16^ Measurements of arm circumference were made across the metacarpal joint at the hand, at the radial styloid and every 10 cm proximal to that point. Relative arm circumference was defined as ipsilateral circumference/contralateral circumference × 100. A value of >110 % was taken to indicate the presence of lymphedema.^6^ The Constant score quantifies overall disability of the arm and shoulder by combining assessments of pain, activities of daily living, range of motion, and power in a single metric with a maximum score of 100.^17^ This score has been used in breast cancer research and has well-defined reliability and validity.^18^,^19^ Quality of life was assessed using the FACT-B+4 and the SF-12 Health Survey (SF12).^20^,^21^ The FACT-B+4 is a comprehensive, breast cancer specific questionnaire that incorporates 5 domains (concerns specific to patients with breast cancer, and physical, social, emotional, and functional well-being). The SF12 is a well-validated, generic, measure of quality of life. Details of the surgery, chemotherapy, and/or radiation therapy were recorded.

### Sample Size Estimate

Previous research demonstrated that ~25–60 % of patients have pain 1 year following breast cancer surgery.^2^–^5^ We took a midrange estimate of 40 % and considered a 20 % absolute reduction in the prevalence of pain as clinically important. Using a 2-sided test at the 5 % level of significance, a sample of 82 patients per group would yield 80 % power to detect a difference of this magnitude. Anticipating a 10 % drop-out rate, we proposed a final sample size of 91 per group.

### Interim Analysis

In July 2011 the Data Safety Monitoring Board performed an interim analysis of blinded, aggregate data. No safety issues were identified among the 66 participants enrolled to this point; however, only 7 participants (11 %, 95 % exact confidence limits 4.4–20.6 %) reported PPP 1 year following surgery. Detection of the specified 20 % absolute reduction in the primary outcome was therefore impossible. Thus, recruitment was stopped, and all enrolled patients were followed to 1 year to provide an estimate of the primary outcome and permit an exploration of secondary outcomes.

### Statistics

Demographic characteristics were summarized for the treatment and control group using means and standard deviations for continuous measures (or medians and interquartile ranges in the case of skewed distributions) and frequencies and proportions for categorical measures. The primary outcome (proportion of patients with arm pain NRS >3 at rest or with arm movement at 1 year) was analyzed using Fisher exact test. Continuous secondary outcomes were assessed for normality and compared at 1 year using 2-sample *t* tests or 2-sample Wilcoxon tests, while categorical outcomes were assessed using Pearson Chi squared tests. SAS v 9.2 was used for all analyses. A 2-sided 5 % level of significance was used for all statistical tests.

## Results

**Fig. 1:**
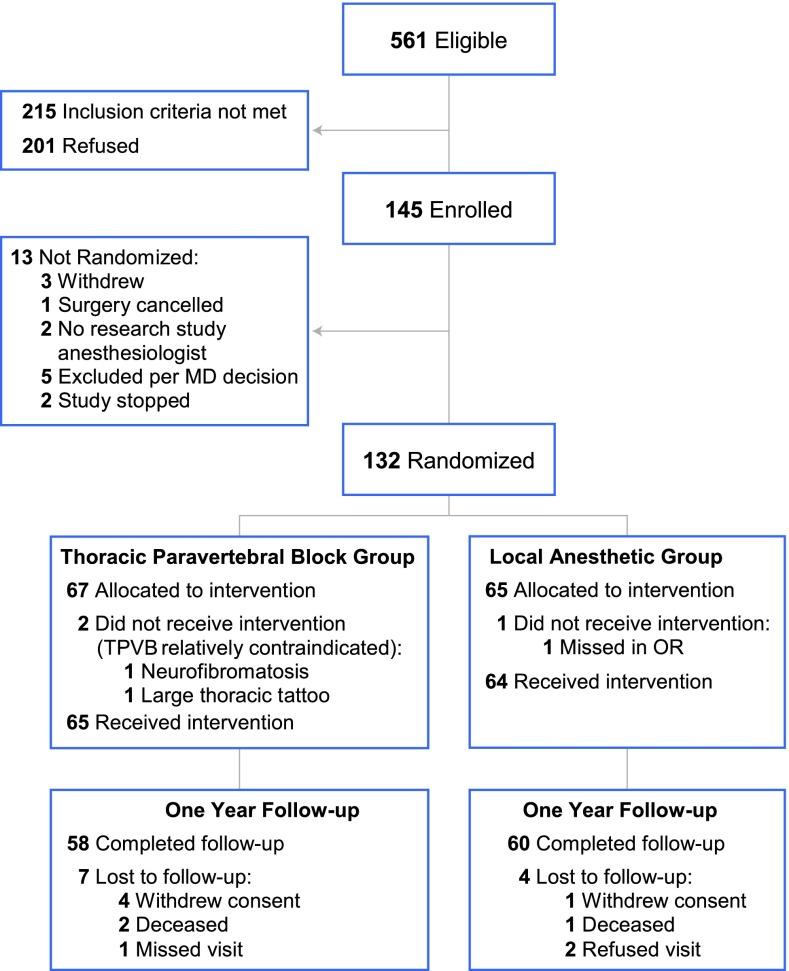
CONSORT diagram showing patient eligibility, enrollment, randomization, and follow-up

Trial recruitment began December 6, 2007 with the last measurement concluded September 28, 2012, A total of 145 participants were recruited; of these, 65 were randomized to the TPVB group and 64 to the LA group. Participant flow is documented in a CONSORT diagram (Fig. 1).

The groups were similar with respect to demographic and treatment characteristics (Table 1). The majority of patients in both groups underwent MRM or simple mastectomy with SNLB (Table 2). There were no surgical complications that required reoperation. The majority of patients in both groups received postoperative chemotherapy and radiation therapy. The dosing and distribution of radiation therapy and number of treatments were similar between groups.

**Table 1:** Preoperative patient characteristics (mean, SD)

Characteristic	TPVB (*N* = 65)	LA (*N* = 64)
Age	54 (10.8)	56 (10.6)
BMI	28.4 (6.5)	29.0 (5.2)
ASA		
1	11 (17 %)	3 (5 %)
2	36 (55 %)	42 (65 %)
3	18 (28 %)	19 (30 %)
Neoadjuvant hormonal therapy	4 (6 %)	4 (6 %)
Neoadjuvant chemotherapy	14 (22 %)	10 (16 %)
Medical comorbidities	31 (48 %)	23 (36 %)
Fibromyalgia	2 (3 %)	0 (0 %)
Anxiety disorder	3 (5 %)	2 (3 %)
Depression	2 (3 %)	3 (5 %)
Diabetes mellitus	7 (11 %)	8 (13 %)
Hypertension	21 (32 %)	20 (31 %)
Peripheral vascular disease	1 (2 %)	0 (0 %)
Hypercholesterolemia	7 (11 %)	5 (8 %)
Stroke/TIA	0 (0 %)	0 (0 %)
Small vessel vasculitis	1 (2 %)	0 (0 %)
Smoker	8 (13 %)	7 (12 %)
Constant score		
Ipsilateral arm	85.47 (9.18)	87.70 (7.37)
Contralateral arm	87.60 (8.28)	88.87 (6.02)
SF-12 MCS	45.62 (11.16)	47.74 (9.69)
SF-12 PCS	52.54 (10.76)	51.60 (8.57)

*BMI* body mass index, *ASA* American Society of Anesthesiologist, *TIA* transient ischemic attack

**Table 2:** Patient surgical and postoperative treatment variables (median, IQR) and (mean, SD)

	Treatment variable		*P* value
	TPVB (*N* = 65)	LA (*N* = 64)	
Surgical variables			
Procedure			0.257
Breast conserving surgery + ALND	9 (14 %)	6 (9 %)	
MRM or Simple mastectomy + SLNB	54 (83 %)	58 (91 %)	
ALND alone	2 (3 %)	0 (0 %)	
Number lymph nodes removed	7 (3, 13)	9.5 (4.5, 19)	0.040
Intercostobrachial nerve preserved	43 (73 %)	27 (50 %)	0.012
Medical oncology variables			
Adjuvant chemotherapy received	41 (63 %)	37 (58 %)	0.507
Radiation oncology variables			
Radiation therapy received	37 (57 %)	42 (66 %)	0.310
Total dose	48.1 (2.90)	49.0 (2.00)	0.193
Total dose per fraction	2.09 (0.20)	2.04 (0.11)	0.192
Site of radiation			
Breast or chest wall	37 (57 %)	42 (66 %)	0.310
Breast or chest wall + lymph nodes	31 (48 %)	38 (59 %)	0.184

*ALND* axillary lymph node dissection, *SLNB* sentinel lymph node biopsy, *MRM* modified radical mastectomy

Chronic pain, arm disability, and quality of life data are shown in Table 3. Only 9 patients (8 %; 95 % CI 4–14 %) met criteria for PPP 1 year following surgery; 5 in the TPVB group and 4 patients in the LA group. Since the trial was not powered to detect differences in these outcomes, we focused on clinically important rather than statistically significant differences between groups. Median BPI severity and interference scores were low in both groups. Both groups showed a small decline in Constant scores. There were 6 patients in the TPVB group and 15 in the LA group who had a 10 % relative increase in arm circumference on the side of surgery, suggesting an overall 18 % incidence of lymphedema. Although the absolute difference between groups was 15 % and statistically significant, this result should be interpreted with caution: (1) multiple testing increases the risk of spurious statistical significance, and (2) the confidence interval around the difference is relatively wide (95 % CI 1.2–28.1 %). Shoulder range of motion (ROM), FACT-B+4, and SF-12 Mental (MCS) and Physical (PCS) Component scores were comparable in both groups.

**Table 3:** Summary of primary and secondary outcomes by groups at 1 year (median, IQR) and (mean, SD)

Assessment tool	TPVB (*N* = 58)	LA (*N* = 60)	*P* value
Primary outcome			
Chronic pain	5 (9 %)	4 (7 %)	0.741
Secondary outcome			
BPI pain severity	0.125 (0–2.25)	0.25 (0–1.50)	0.989
Pain interference	0 (0–1)	0 (0–0.86)	0.744
Opioids taken	1 (2 %)	1 (2 %)	1.000
Constant score			
Change from baseline (ipsilateral arm)	−5.23 (15.02)	−7.23 (13.53)	0.448
Change from baseline (contralateral arm)	−2.25 (13.57)	−3.01 (11.60)	0.809
FACT-B+4^a^	138.5 (116, 148.75)	139.5 (125.38, 151.5)	0.683
SF-12 MCS (change from baseline)	6.06 (9.86)	4.62 (9.28)	0.417
SF-12 PCS (change from baseline)	−4.66 (12.96)	−4.83 (9.22)	0.934
Arm lymphedema	6 (10 %)	15 (25 %)	0.038
Shoulder range of motion			
Ipsilateral arm—degrees forward	160 (150, 160)	160 (140, 160)	0.388
Ipsilateral arm—degrees abduction	160 (155, 160)	160 (147.5, 160)	0.485
Contralateral arm—degrees forward	160 (160, 160)	160 (159.5, 160)	0.324
Contralateral arm—degrees abduction	160 (160, 160)	160 (154, 160)	0.612

*SF*-*12 MCS* SF-12 mental component summary, *SF*-*12 PCS* SF-12 physical component summary

^a^Only recorded postoperatively

Characteristics of the 9 patients with chronic pain at 1 year are shown in Table 4. These patients had a median of 14 (IQR, 7.0–19.0) lymph nodes removed, and 3 patients (33 %) had their intercostobrachial nerve preserved. Lymphedema was common as was a large decrease in the Constant score in the ipsilateral arm. Lowered quality of life was reflected in FACT-B+4 and SF-12 PCS scores. Despite pain and swelling, ROM of both arms was preserved and no patient required opioid analgesia.

**Table 4:** Outcomes of patients with persistent postoperative pain (median, IQR) and (mean, SD)

Assessment tool	Patients with pain (*N* = 9)
BPI Pain severity	3.25 (2.75–4.25)
Pain interference	4.57 (2.14–5.00)
Constant score	
Change score from baseline (ipsilateral arm)	−24.69 (18.01)
Change score from baseline (contralateral arm)	−4.24 (17.73)
FACT-B+4	109.00 (92.00, 120.00)
SF-12 MCS (change from baseline)	9.50 (10.56)
SF-12 PCS (change from baseline)	−17.14 (18.34)
Arm lymphedema	6 (67 %)
Shoulder range of motion	
Ipsilateral arm—degrees forward	160 (130, 160)
Ipsilateral arm—degrees abduction	160 (120, 160)
Contralateral arm—degrees forward	160 (160, 160)
Contralateral arm—degrees abduction	160 (160, 160)

## Discussion

### Incidence of Persistent Postoperative Pain

Only 8 % of women in this trial reported PPP 1 year following major breast cancer surgery; much lower than the 25–60 % reported in the literature. Because of the surprisingly low incidence of PPP, we were unable to compare the efficacy of TPVB and LA. Possible explanations for this discrepancy are: study design, perioperative surgical care, perioperative anesthesia care, and definition of PPP. The majority of studies on PPP following breast surgery are conducted retrospectively via questionnaire, ~2–2.5 years and up to 3.5–4 years following surgery.^5^,^22^–^25^ The retrospective questionnaire study design and long duration since the inciting event, increase the possibility of error. In contrast, patients in our study were prospectively assessed by a trained nurse at each follow-up visit.

Intercostobrachial neuralgia is a recognized etiology of chronic pain following breast cancer surgery.^2^ It has been recommended that for optimal quality of cancer care, “not only treatment of tumours with a low incidence but also other complex or high risk cancer procedures should be provided in a specialized setting, with the right infrastructure, sufficient volume and adequate expertise.”^26^ All surgeons involved in this study have subspecialized practices focusing on breast cancer surgery. Although no studies have been done to directly examine the relationship between surgical expertise and pain outcomes, Kehlet^27^ suggests that “nerve injury might be reduced by surgical expertise,” thus reducing the risk of PPP. Indeed in the majority of our patients, the intercostobrachial nerve was identified and preserved. The median number of lymph nodes removed in our study is on the lower end of the range reported by Olaya et al.^28^ who examined the number of nodes removed in current surgical practice. These authors noted that “increased breast surgery practice is associated with a decreased number of (nodes) removed.” Since there is a proportional relationship between the number of lymph nodes removed and the occurrence of PPP following breast cancer surgery, the low number of nodes removed is in keeping with our speculation that surgical factors in our study may have contributed toward improved pain outcomes.^29^

The intensity of acute postoperative pain has been identified as a predisposing risk factor to the development of PPP.^23^,^30^,^31^ The anesthetic techniques used in this study incorporated multimodal, procedure-specific techniques designed to minimize postoperative pain. Both TPVB and LA infiltration have been shown to provide superior analgesia in breast surgery compared with opioid medications alone.^4^,^9^ Mitchell et al.^32^ showed that co-analgesics acetaminophen + ibuprofen were as effective as Tylenol 3 in treating pain in breast cancer patients. We preemptively treated and postoperatively prescribed to patients a long (7-day) course of co-analgesic medications that have been shown to contribute to improved postoperative analgesia.^33^ The synergism of multimodal pain regimens using TPVB or LA infiltration, in combination with effective co-analgesics and meticulous surgical practices, likely contributed to our low incidence of PPP.

There is no agreement in the literature on the definition of PPP; many studies use subjective definitions.^34^ Grigoras et al.^35^ prospectively studied 36 breast cancer patients receiving perioperative intravenous lidocaine versus placebo and found an overall 3-month incidence of PPP of 31 %; however, they subjectively defined PPP as an affirmative answer to “Have you had pain in the last week which you attribute to your breast surgery?”. Fabro et al.^29^ reported prospective data collected 6 months following breast cancer surgery and found a 52 % incidence of pain. These investigators also defined pain subjectively using reports of hyperesthesia and percussion tenderness. Neither author used established and validated measurement tools to evaluate for the presence of pain. We used multiple, robust measures of pain intensity in our study and considered it important to use a meaningful definition of PPP that has functional significance to the patient. A NRS >3 signifies pain that impacts a patient’s mood and activity and is a reliable, well established definition of clinically relevant moderate to severe pain in cancer patients.^36^–^38^ Our patients’ low BPI pain severity scores and low interference scores corroborate our low rate of PPP.^37^,^38^

### Arm Morbidity and Quality of Life

Patients in our study showed impairments in ROM classified as “mild” when compared with age and gender norms^39^ Our 18 % overall incidence of lymphedema is comparable to that reported in the literature as are our patients’ SF-12 and FACT-B+4 scores.^40^–^42^ It should be noted, however, Constant scores were reduced, lymphedema was more common, and quality of life was poor among the 9 women with PPP. Indeed, their 1-year SF-12 PCS was similar to that reported by patients with chronic heart, lung, and kidney disease and their FACT-B+4 scores were lower than those reported in the ALMANAC study, indicating a severe, disease-specific burden of illness.^42^,^43^ Clearly PPP is a significant problem for those affected.

### Study Limitations

Our study was stopped early following interim analysis. Expanding to a multicenter study may have allowed for increased patient recruitment. Because of our low incidence of PPP, we had insufficient events to allow for identification of predictors for PPP.

## Conclusions

This prospective study reports an 8 % incidence of PPP 1 year following major breast cancer surgery with lymph node resection. Our patients experienced minor declines in arm function, and quality of life did not diminish. Patients can be reassured that experienced surgical teams dedicated to breast oncology who use multimodal analgesic therapies may achieve low rates of PPP and preserve both activity and quality of life 1-year following breast surgery. Although PPP was not frequent at 1 year, it did have a large impact on the affected patients’ arm morbidity and quality of life. Future avenues of research should continue to focus on ways to reduce and treat PPP and arm morbidity in women undergoing breast cancer surgery.
